# Therapeutic hypothermia in ECPR: re-examining neuroprotection in refractory cardiac arrest

**DOI:** 10.1186/s40635-025-00845-6

**Published:** 2025-12-23

**Authors:** Balasubrahmanyam Chandrabhatla

**Affiliations:** Citizens Specialty Hospital, Nallagandla, Hyderabad, India

Refractory cardiac arrest remains one of the most difficult conditions in critical care, with neurologically intact survival rates still low despite advances in systems of resuscitation. Extracorporeal cardiopulmonary resuscitation (ECPR) has expanded the boundaries of salvageability by providing immediate circulatory support, yet hypoxic–ischemic brain injury continues to dominate long-term outcomes. Optimizing neuroprotection within this unique physiological setting is therefore essential.

In this issue, the study by Anthony Moreau et al.,*“Effects of therapeutic hypothermia on brain function in a refractory cardiac arrest model treated with extracorporeal cardiopulmonary resuscitation”*, offers timely mechanistic insight into one of the most debated aspects of post-arrest care—therapeutic hypothermia, or targeted temperature management (TTM). Their large-animal ECPR model provides high-resolution physiological and histological data that clarify how hypothermia may retain relevance in the era of extracorporeal reperfusion.

## Therapeutic hypothermia: a question reopened

Major randomized trials such as TTM [1] and TTM2 [2] have challenged the benefit of deeper hypothermia after cardiac arrest, showing little difference between 33 °C and 36 °C, or controlled normothermia. However, these studies included minimal ECPR representation and cannot be extrapolated to extracorporeal reperfusion. The physiology of ECPR—rapid, controlled restoration of circulation with predictable reperfusion timing—differs fundamentally from conventional CPR and may uniquely amplify the neuroprotective potential of hypothermia by modifying reperfusion injury dynamics [3].

Using a sophisticated porcine ECPR model, Moreau et al. report:

- *Faster neurophysiologic recovery*. Earlier return of organized EEG and improved amplitude-integrated EEG patterns in cooled animals—parameters strongly associated with neurological outcome.
- *Reduced biochemical injury*. Lower NSE and GFAP levels support attenuation of neuronal and glial damage.
- *Stabilized cerebral perfusion*. Hypothermia reduced post-reperfusion hyperemia and improved cerebral oxygenation.
- *Structural preservation*. Histopathology revealed less cortical and hippocampal injury in the hypothermia group.

Collectively, these findings suggest that hypothermia may retain meaningful neuroprotective effects specifically within the controlled environment of ECPR, even as its value appears reduced in non-extracorporeal cardiac arrest. Clinical translation must be cautious, but the mechanistic signals align with the established effects of hypothermia [4]: reduced cerebral metabolic demand, mitochondrial stabilization, modulation of inflammatory and excitotoxic pathways, and preservation of blood–brain barrier integrity. These benefits mirror its proven role in cardiac surgery and neonatal encephalopathy. Importantly, ECPR offers a precise reperfusion time point, enabling early, targeted cooling at a biologically optimal moment.

The study highlights critical priorities for advancing ECPR-specific neuroprotection:

- *Optimal temperature targets.* Tailored to extracorporeal physiology.
- *Therapeutic timing.* Defining how early cooling must begin relative to cannulation.
- *Phenotype-specific responses.* Informed by arrest etiology and downtime.
- *Integration of multimodal neuromonitoring* (EEG, NIRS, biomarkers, imaging).
- *Combination strategies.* Including controlled reperfusion and metabolic modulation.

Dedicated multicentre trials focusing exclusively on ECPR patients are urgently needed, with emphasis on high-quality neurological outcomes and mechanistic endpoints.

Moreau et al. [5] deliver compelling preclinical evidence that therapeutic hypothermia shows promising neuroprotection in ECPR, warranting dedicated trials to refine temperature targets and guide personalized post-arrest care.

## Data Availability

This editorial does not involve the generation or analysis of new experimental data. All scientific information discussed—including findings related to therapeutic hypothermia, neuroprotection, and extracorporeal cardiopulmonary resuscitation (ECPR)—is derived from previously published studies and publicly accessible sources. These works are fully cited throughout the manuscript. No new datasets were created, and therefore no additional data are available for sharing.
